# Energy allocation and behaviour in the growing broiler chicken

**DOI:** 10.1038/s41598-018-22604-2

**Published:** 2018-03-14

**Authors:** Peter G. Tickle, John R. Hutchinson, Jonathan R. Codd

**Affiliations:** 1 School of Biomedical Sciences, Faculty of Biological Sciences, University of Leeds, Leeds, UK; 2 Department of Comparative Biomedical Sciences, Structure and Motion Laboratory, Royal Veterinary College, London, UK; 3 School of Biological Sciences, Faculty of Biology, Medicine & Health, University of Manchester, Manchester, UK

## Abstract

Broiler chickens are increasingly at the forefront of global meat production but the consequences of fast growth and selection for an increase in body mass on bird health are an ongoing concern for industry and consumers. To better understand the implications of selection we evaluated energetics and behaviour over the 6-week hatch-to-slaughter developmental period in a commercial broiler. The effect of posture on resting metabolic rate becomes increasingly significant as broilers grow, as standing became more energetically expensive than sitting. The proportion of overall metabolic rate accounted for by locomotor behaviour decreased over development, corresponding to declining activity levels, mean and peak walking speeds. These data are consistent with the inference that broilers allocate energy to activity within a constrained metabolic budget and that there is a reducing metabolic scope for exercise throughout their development. Comparison with similarly sized galliforms reveals that locomotion is relatively energetically expensive in broilers.

## Introduction

Production of broiler chickens is a major component of the global need for affordable sources of protein, with current UK annual production alone accounting for over 1000 million birds (DEFRA Statistics, 2017). Intensive genetic selection over the past 60 years for desirable performance traits such as rapid growth, large pectoral muscles and high feed-conversion efficiency has engineered a modern industrial bird with strikingly different characteristics to the ancestral *Gallus gallus*. The results of breeding programmes on agricultural production are considerable but there are also unintended consequences for broiler health and welfare. Growing birds can be afflicted by a range of pathological conditions, including diseases of the locomotor^1–5^ and cardio-pulmonary systems^6–8^ and heightened susceptibility to infection^9^. Broiler health concerns influence not only the birds’ welfare but also represent an appreciable economic loss to the industry through premature mortality and carcass rejection at processing. Therefore the modern broiler is a remarkable success story whereby the natural characteristics of the chicken have been harnessed to maximise meat production, but welfare concerns related to rapid growth and high body mass persist.

There is mounting evidence to trace the causal mechanisms of these health problems back to selection for large pectoral muscle growth and rapid increase in body mass. Intensive selective breeding has engineered a fourfold increase in broiler growth rate, reducing the time-to-slaughter from 16 to 6 or fewer weeks^10–12^. A corresponding increase in breast muscle yield has culminated in pectoral mass accounting for approximately 20% of body mass in the modern bird, double the size in ancestral varieties^13–15^. Experimental manipulation of pectoral mass in birds indicates that as mass increases, the energetic costs associated with locomotion disproportionately increase^16,17^, likely due to an increase in the energetic resources required to power inspiratory and expiratory dorsoventral movements of the ribs and sternum^18–20^. Increased metabolic cost associated with moving a disproportionately heavy sternum (including pectoral muscle) has implications for the pectoral hypertrophy in broilers because, as sternal mass increases, ventilatory movements may become more expensive and/or respiratory capacity may decline. Circumstantial evidence for this can be found in reports of pulmonary disease in heavy broilers, which is associated with heart failure and mortality^21,22^.

Skeletal development also lags behind pectoral growth, potentially imposing a functional limitation on the efficacy of the breathing apparatus. For example the uncinate processes, key respiratory structures in birds that facilitate inspiration and expiration^20,23^, remain incompletely ossified even at slaughter weight^15^. As these processes act as levers for movement of the ribs and therefore sternum during breathing, the lack of ossification, which stiffens the processes, will influence their action^20^. A consequent relative reduction in respiratory capacity can be linked to behavioural changes, because oxygen delivery is a key determinant of locomotor performance^24^. Developing birds follow a trend for increased time spent resting and decreased locomotor activity^25,26^, indicative of reduced locomotor stamina.

Unfortunately, few data are currently available on the energetics of breathing and locomotion in modern broilers^27^ and the impact on development of pathology is equivocal. It is important therefore to establish how body morphology, locomotor, ventilatory and metabolic factors are linked and to determine how selection for rapid growth and muscle hypertrophy influences the balance of these interactions in broilers. Therefore, our primary objective was to develop an understanding of the interactions between developmental stage, locomotor activity and energetic aspects of broiler growth. Dramatic changes in morphology in a short developmental period allow evaluation of whether declines in performance (i.e. ventilatory, energetic or biomechanical) occur simultaneously, and if there are specific time points that are associated with imposition of constraints. We hypothesized that decreasing activity levels are associated with increased resting and locomotor metabolic costs. A better understanding of these underlying physiological factors may potentially inform future strategies to produce healthier birds and enhance production standards.

## Methods

### Birds

Commercial broilers (Cobb® 500) were procured from a local supplier, housed in pens, provided with *ad libitum* access to poultry pellets and water throughout and weighed daily to monitor growth and general health. When multiple broilers were observed simultaneously, similar-sized birds of the same age were used. Furthermore, any birds that were lame or showing symptoms of ‘leg weakness’ were not included in studies of metabolism or activity as assessed by established gait scoring protocol^28^. Experimental procedures and methods were carried out under ethical approval from the University of Manchester Ethics Committee in accordance with the Animal (Scientific Procedures) Act 1986, covered by a Home Office project licence (40/3549) held by Dr Codd.

### Behaviour

Birds were observed in a specially constructed arena (dimensions: 1.2 m × 1.2 m; height of the wooden sides of the arena: 0.33 m). Food and water were available to the birds inside the arena. Black rubber matting was used as the floor material in the arena to enhance the colour contrast between the floor and birds. In each experiment, five birds were placed in the arena after which the handler left the room. Birds were filmed at 25Hz using a Sony Handycam mounted on a tripod overlooking the arena. The initial 20 minutes of footage was discarded to limit the confounding effects of handler interference on bird behaviour. Videos were subsequently converted into a low-resolution greyscale format to enhance accuracy of the analysis software (Lolitrack, Loligo Systems, Denmark). Birds were automatically assigned an identifying number in the opening frame and the position of each individual was tracked frame-by-frame, enabling calculation of overall activity, speed and distance moved. Occasional the automatic system would misidentify individuals when birds clustered together, which resulted in a removal of contrast between background and each individual bird. These instances were identified by the programme and manually corrected by re-assigning the original ID number according to distinguishing markings on the relevant birds. Video files were calibrated with a scale bar to allow determination of accurate velocities and distances. Tracking software was optimised in each case through a process of varying image contrast and marker size (i.e. number of pixels tracked). A video file displaying tracked birds was generated in each case, facilitating manual assessment of tracking; occasional erroneously placed markers were corrected at this stage. Coordinate data from each computer-tracked bird was used to calculate distance travelled, average and peak velocities. The time broilers spent actively moving was logged and converted to a proportion of total experimental period.

A total of 9 multi-bird trials (8 trials consisting of 5 birds and 1 trial of 3 birds) were conducted. Duration of the analysed period of activity typically lasted 59.85 ± 3.55 minutes (mean ± standard error). Broken-line regression models were fitted between behavioural variables and body mass (Mb) if a significant change in regression slope was detected using Davies’ test^29^. This approach calculated whether there is a threshold value at which two significantly different regression lines are connected, allowing a calculation of whether there is a ‘tipping point’ across broiler development. Breakpoint analyses were conducted using the segmented package^30^ in R (version 3.1.0).

In order to determine if the laboratory data were comparable to birds housed commercially, complimentary experiments were conducted in a farm setting, whereby behaviour of 5 randomly selected birds was monitored within a wire-fence pen. Birds were a commercial variety being grown for slaughter. Total flock size within the shed (2512.6 m^2^) at chick placement (i.e. day after hatch) was 47960. Within the shed a small penned area (0.78 m × 1.56 m; 1.22 m^2^) was constructed using metal fencing; a drinker ran through the centre of the pen and a feeder was provided (identical to the one used in the laboratory experiments). The remaining flock was free to walk around the pen and interact with the birds inside, as the metal fencing did not prevent visual, olfactory or auditory communication. A tripod was positioned over the pen to allow mounting of a video camera (Sony Handycam, 25 Hz) for filming. The initial 20 minutes of footage was discarded to minimize the effect of handler on bird behaviour. Footage was divided into 20-second periods, in which the predominant activity state of each bird was logged; sitting, standing or active. ‘Active’ refers to periods when the birds were moving inside the chamber. Data were pooled in each trial to give an overview of behavioural state within a group of birds. Duration of the analysed period of activity typically lasted 51.57 ± 6.43 minutes (mean ± standard error).

### Respirometry

In all cases 100ml min^−1^ of excurrent gas was sub-sampled, drawn through a water vapour meter (RH-300: Sable Systems, Las Vegas, NV, USA) and carbon dioxide analyser (FoxBox: Sable Systems, Las Vegas, NV, USA). The airstream was dried using a column of magnesium perchlorate prior to measurement of CO_2_. Metabolic rate was determined as the rate of CO_2_ production using equation 10.5 from Lighton^31^:

1 $${\dot{V}}_{{{\rm{CO}}}_{2}}=({{\rm{FeCO}}}_{{\rm{2}}}-{{\rm{FiCO}}}_{2}\ast {\rm{FR}}/(1-{{\rm{FeCO}}}_{{\rm{2}}}\ast (1-(1-{\rm{RER}})))$$

where FiCO_2_ and FeCO_2_ are the proportions of CO_2_ in air entering and leaving the respirometry chamber. FR is the main flow rate of air into the chamber after mathematical correction for the diluting effects of water vapour (equation 8.6 in^31^). RER is the respiratory exchange ratio (V˙CO_2_: V˙O_2_). Since oxygen consumption was not measured, an RER of 0.85 was assumed in all experiments to minimize the error in calculations of metabolic rate^32^. Data analysis was conducted in ExpeData (Sable Systems, USA).

#### Single bird

Flow-through respirometry was used to determine resting metabolic rate (RMR). Food was available until the onset of each experiment, and consequently the energetic contribution of digestion is included in estimates of RMR. In each experiment, a broiler was selected at random and placed into a chamber (volume 61 L) from which air was drawn at 50 L min^−1^. The experimental period consisted of the bird quietly resting while V˙CO_2_ was simultaneously recorded. An observer noted when standing and sitting postures occurred. Ambient CO_2_ levels were recorded before and after measurement of the bird to allow for correction of any change in room-air composition. Laboratory conditions (temperature, humidity and light) were maintained to control factors potentially affecting choice of resting posture^33^. The most stable 60-second V˙CO_2_ plateau was considered representative of RMR in each posture. The birds showed no temporal preference for resting posture, and consequently sitting and standing occurred in no particular order. Data from 79 experiments (using 41 individuals; some birds were resampled at later developmental stages) were analysed. The relationship between increasing body mass and RMR in sitting (RMR_sit_) and standing (RMR_stand_) postures was determined using linear regression.

#### Multiple birds

Protocol: Assessment of active metabolic rate (AMR) is difficult because broilers lack the stamina and/or inclination for treadmill locomotion. Therefore, we developed a novel indirect method to estimate metabolic rate during activity by coupling behavioural and gas analysis techniques. In essence, the single-bird respirometry protocol was modified to include gross measurement of multiple birds in a single, enlarged respirometry chamber. Excurrent air sampled from the experimental apparatus was consequently a mixture of respiratory gases from multiple birds, arising from metabolic processes during rest and activity. Integration of matched behavioural and energetic data derived from multiple birds and the results of single bird respirometry allowed us to mathematically infer the energetics of resting and locomotion.

The arena to determine activity patterns and locomotor parameters was modified by incorporating a transparent sheet of Perspex, which was supported by the pre-existing side panels to form a roof. The arena was slightly enlarged to dimensions of 1.75 m × 1.2 m × 0.33 m. The increased size and addition of a transparent roof allowed conversion into a large (700 L) respirometry chamber. Food and water were provided within the chamber to mimic farm conditions. A cross-shaped arrangement of pipework emanating from a central 4-way connector was suspended from the Perspex roof. This network of pipes was connected to a mass flow pump (FlowKit Mass Flow Generator; Sable Systems, Las Vegas, USA) operating at a flow rate of 500 L min^−1^. Holes were drilled along each branch to allow air from throughout the chamber to be quickly sampled. The 3 open ended pipes were sealed to ensure that air was drawn through the holes (n.b. the fourth branch of the arrangement was connected to the mass flow pump), thereby sampling across the chamber simultaneously. Accuracy of this system was determined by flowing a known amount of nitrogen into each corner of the chamber using a mass flow controller (MFS; Sable Systems, Las Vegas, USA). The inaccuracy of our observed value compared to the expected value for nitrogen dilution averaged 5.5%, corresponding to values reported for smaller respirometry chambers, e.g.^34,17^. The time delay between introduction of nitrogen and deflection of CO_2_ value was determined and applied in the analyses of respirometry data; typically changes in gas composition due to animal introduction were detected in approximately 1 minute.

Groups of 5 birds were introduced into the chamber, after which the handler left the room. Behaviour was determined using a video recording taken from an overhead perspective. Videos were synchronized with the gas data so that behavioural state and metabolic rate were aligned. Data from the initial 20 minutes of each experiment were discarded to remove for any confounding effects of handling on bird behaviour and metabolic rate. Since the respiratory gas composition of more than one animal was sampled concurrently, any confounding effect of differences in body mass were limited by selecting similarly sized birds (see supplementary data).

Data analysis: The behavioural status of each bird was manually assigned by an observer from the video recordings. Videos were was divided into 20-second periods and the predominant activity state of each bird was logged; sitting, standing or active. ‘Active’ refers to periods when the birds were moving inside the chamber. Consequently, in each minute of footage, 5 birds each had 3 measurements, corresponding to a total of 15 behavioural measurements. Respirometry data were smoothed in ExpeData by averaging into 1-minute bins. Using the video recordings, periods comprising a minimum of 5 minutes when all birds were sitting quietly were identified. Corresponding V˙CO_2_ data for this period provided a direct measure of RMR_sit_. For each bird in the chamber, the magnitude of RMR_stand_: RMR_sit_ was calculated according to body mass using the relationship determined in our analysis of postural energetics. A mean value of this ratio was then used to transform RMR_sit_, into predicted RMR_stand_. A net cost of standing, i.e. the RMR increment in excess of RMR_sit_, was then calculated as:

2 $${{\rm{RMR}}}_{{\rm{stand}}\_{\rm{inc}}}={{\rm{RMR}}}_{{\rm{stand}}}-{{\rm{RMR}}}_{{\rm{sit}}}$$

In each 1-minute bin, RMR_sit_ was subtracted from AMR, leaving a remainder, RMR_stand_inc_. We consider RMR_sit_ to be a baseline cost that remains a constituent of metabolic rate during activity and inactivity. RMR_stand_inc_ was then adjusted by the proportion of time in each 1-minute bin spent exhibiting that behaviour and subtracted from the ‘remainder’ to leave only the V˙CO_2_ accounted for by activity. Finally, this value was divided by the fraction of time spent actively moving to give the increment accounted for by activity (AMR_inc;_) in excess of RMR_stand_. In summary, AMR was calculated as:

3 $${\rm{AMR}}={{\rm{RMR}}}_{{\rm{sit}}}+{{\rm{RMR}}}_{{\rm{stand}}\_{\rm{inc}}}+{{\rm{AMR}}}_{{\rm{inc}}}$$

AMR_inc_ was derived when activity accounted for at least 20% of the behavioural recording in each minute, i.e. at least 3 of 15 samples in the behavioural assay. When activity occurred for shorter periods it was difficult to calculate meaningful data due to the relatively small activity-related V˙CO_2_ signal; therefore our calculation may be a conservative estimate of AMR_inc_. Data presented as V˙CO_2_ (ml min^−1^) refer to total group-V˙CO_2_ divided by 5 (number of birds), i.e. V˙CO_2_ per bird. Our methodology for determining the relative contributions of RMR and AMR_inc_ to overall AMR assumes a simple additive relationship (Eq. 3)^35,36^. By determining the relationships between RMR and body mass independently of AMR measurement, we attempted to minimise the effect of statistical non-independence of RMR and AMR_inc_ (since AMR_inc_ is calculated as the remainder of AMR after subtraction of RMR). This approach may be limited due to metabolic processes ongoing during RMR being downregulated when activity occurs^37^, causing an underestimation of broiler AMR_inc_. Nevertheless, the methods implemented in this paper allow us to estimate how energy is allocated in broilers across development.

Calculation of cost of transport (CoT: J kg^−1^ m^−1^) used the relationship between body mass and average walking speed derived in our behavioural tracking experiments. Broiler CoT was calculated from V˙CO_2_ assuming an RER of 0.85 and a conversion of 20.1 J per ml O_2_. It should be noted that our estimate of CoT may be confounded by experimental design; transient bursts of activity, rather than steady-state locomotion are included in our metabolic analysis and this includes the potentially costly elements of acceleration, deceleration and postural changes. Therefore, it was possible that we over-estimated CoT. Scaling analysis using linear regression was performed on log-transformed data assuming an isometric relationship to be proportional to Mb1.00. Comparative data for RMR and CoT in a size-range of adult galliform birds were taken from the literature. Calculation of comparative CoT data relies upon extrapolation of published regression equations to walking speeds slower than were actually measured, because broilers walk at a slower pace than birds in previous treadmill-based studies. Any error potentially linked to this approach is likely to be small due to the highly significant relationships between walking speed and metabolic rate in galliforms. The regression intercept of these equations was assumed to represent RMR, as true RMR values are not available in all cases. The ANCOVA function in the PAST statistical program^38^ was used to determine whether regression equations were significantly different.

## Results

### Behaviour

**Figure 1:**
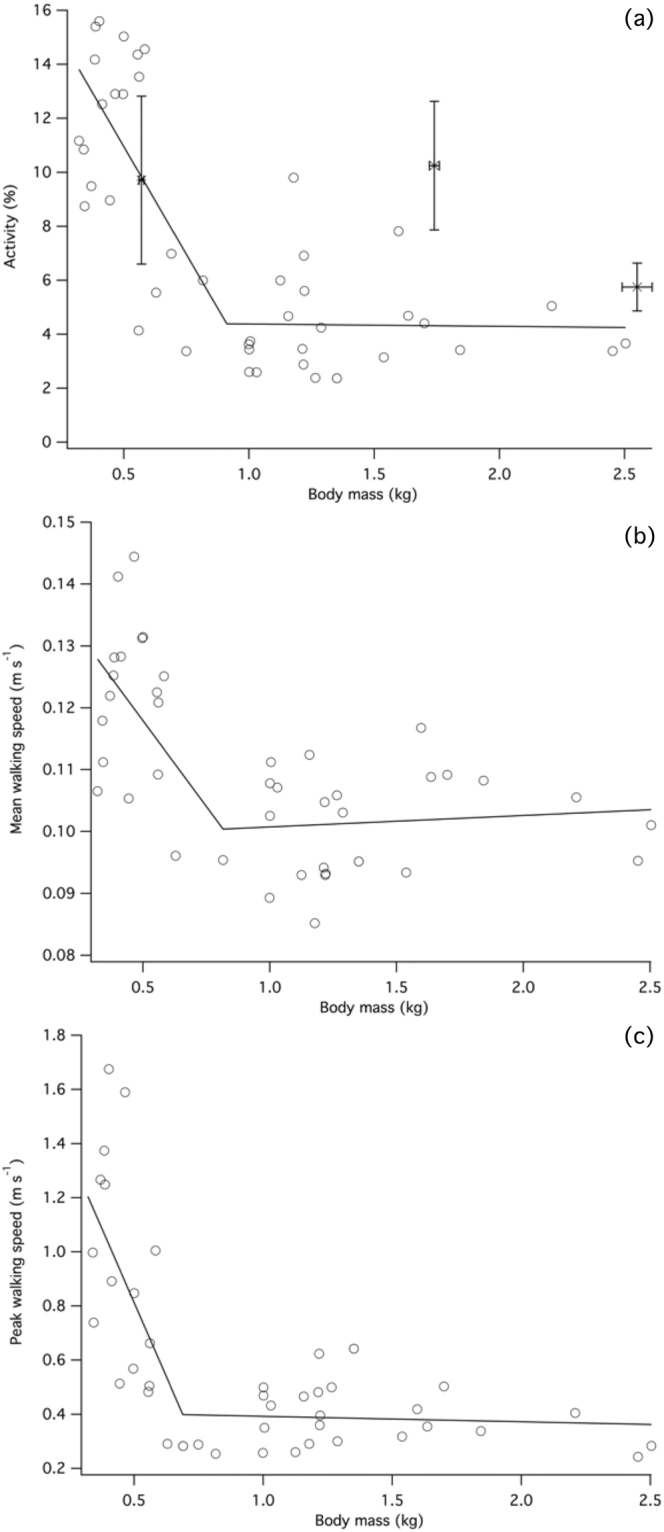
Broiler behavioural changes over development. (**a**) Rapid decline in activity occurred between 0.321 and 0.911 kg (y = −15.98x + 18.940) while no further decreases were detected in heavier birds (y = −0.08702x + 4.465). Activity ± SEM for data collected on a commercial farm is denoted by asterisks. (**b**) Mean walking speed decreased until broilers reached 0.816 kg (y = −0.05549x + 0.1457), after which no further significant decline in speed was detectable (y = −0.001872x + 0.09885). (**c**) Peak walking speed initially decreased rapidly (y = −2.184x + 1.906) followed by a slower decline (y = −0.01996x + 0.4126).

Large changes in behaviour were apparent over the growth period, as periods of activity and walking speed rapidly declined as the birds aged. Proportion of time spent actively moving significantly decreased over development (Fig. 1a), from 12.06% ± 0.73 in birds weighing less than 0.5kg to 4.03% ± 0.52 in broilers over 2.0 kg. Changes in behaviour were correlated with increasing body mass until a breakpoint value of 0.911 ± 0.135 kg (Davies’ test P < 0.001), after which further declines in performance were negligible. Two regression lines were fitted as declining activity occurred between 0.3 and 0.91 kg (y = −15.98x + 18.940, slope 95% CI ± 9.385) whereas no further decreases were detected in heavier birds (y = −0.08702x + 4.465; slope 95% CI ± 2.600).

Behavioural measurements from the farm-based observations indicated a similar pattern of declining activity (Fig. 1a), whereby birds weighing 0.57 kg ± 0.01 were active for 9.71% ± 3.10 compared to 5.75% ± 1.55 in 2.55 kg broilers. This delayed decline in activity, i.e. no decline in birds under 1.74 kg, differed from laboratory-based experiments.

Mean walking speed (Fig. 1b) followed a similar trajectory whereby velocity decreased until body mass attained 0.816 ± 0.125 kg (Davies’ test: P = 0.003; y = −0.05549 + 0.1457, slope 95% CI ± 0.036), after which no further decline was observed (y = 0.001872x + 0.09885, slope 95% CI ± 0.010). Peak walking speed (Fig. 1c) also declined in a two-phase relationship (Davies’ test: P < 0.001), with a steeper decline occurring in birds weighing under 0.690 ± 0.073 kg (y = −2.184 + 1.906, slope 95% CI ± 1.088) than for heavier birds (y = −0.01996x + 0.4126, slope 95% CI ± 0.212).

### Energetics of posture

**Figure 2:**
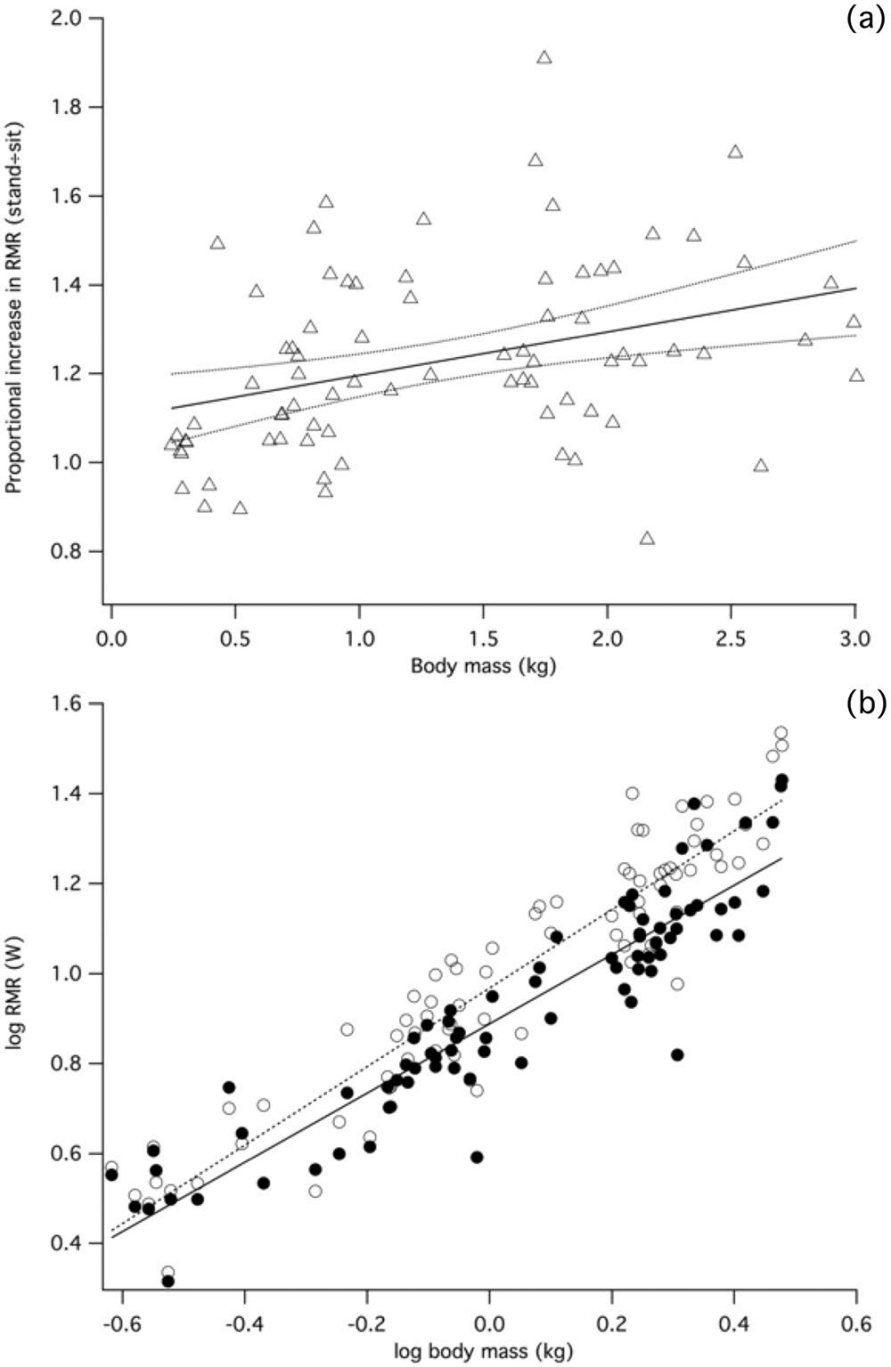
Resting posture influences magnitude of RMR. (**a**) Log – log plot showing the allometric relationship between RMR and body mass. Filled circles, sitting RMR; open circles, standing RMR. Each marker represents mean data from a single experiment in which a broiler exhibited quiet resting in sitting (solid line: y = 1.287x^0.770^; P < 0.001) and standing postures (dashed line: y = 1.367x^0.873^; P < 0.001). (**b**) Ratio of standing/sitting RMR over development in broilers. There is a significant increase in the energetic cost of standing as birds grow (y = 0.098x + 1.098; P = 0.001).

Relative to increasing body mass, RMR_sit_ and RMR_stand_ increased with negative allometry (Fig. 2a, Table 1), i.e. the scaling exponent was below ∝Mb^1.00^ indicating that mass-specific RMR was reduced in larger birds. RMR was significantly influenced by broiler resting posture - as birds grew, RMR_stand_ became progressively more expensive compared to RMR_sit_, indicated by the significantly different regression line slopes (ANCOVA: F = 3.897, P = 0.05). The increased energetic cost relative to RMR_sit_ of RMR_stand_ increased from 12% to 39% as birds grew from 0.2 kg to 3.0 kg (Fig. 2b, Table 1).

**Table 1:** Results of the regression analyses performed on broiler V˙CO_2_ (single and multiple-bird respirometry).

Protocol	y-variable	N	F	t	R^2^	P	intercept	slope	slope 95% CI
Single bird	log RMRsit	79	451.597	21.251	0.854	<0.001	1.287	0.770	0.697–0.842
	log RMRstand	79	528.237	22.983	0.873	<0.001	1.367	0.873	0.797–0.949
	RMRstand/RMRsit	79	11.275	3.358	0.128	0.001	1.098	0.098	0.040–0.156
Multiple birds	RMRsit	10	200.268	14.152	0.962	<0.001	18.072	9.152	4.965–13.337
	AMRinc	10	12.603	3.55	0.612	0.008	11.864	7.428	2.05–12.253
	AMR	10	169.225	13.009	0.955	<0.001	20.319	32.330	26.605–38.060
	log RMRsit	10	130.569	11.427	0.942	<0.001	1.449	0.696	0.55–0.837
	log AMRinc	10	20.702	4.55	0.721	0.002	1.295	0.444	0.219–0.669
	log AMR	10	226.692	15.056	0.966	<0.001	1.736	0.660	0.559–0.761
	AMRinc/AMR	10	8.261	2.874	0.508	0.021	0.444	−0.065	−0.118–0.013

### Energetics of activity

**Figure 3:**
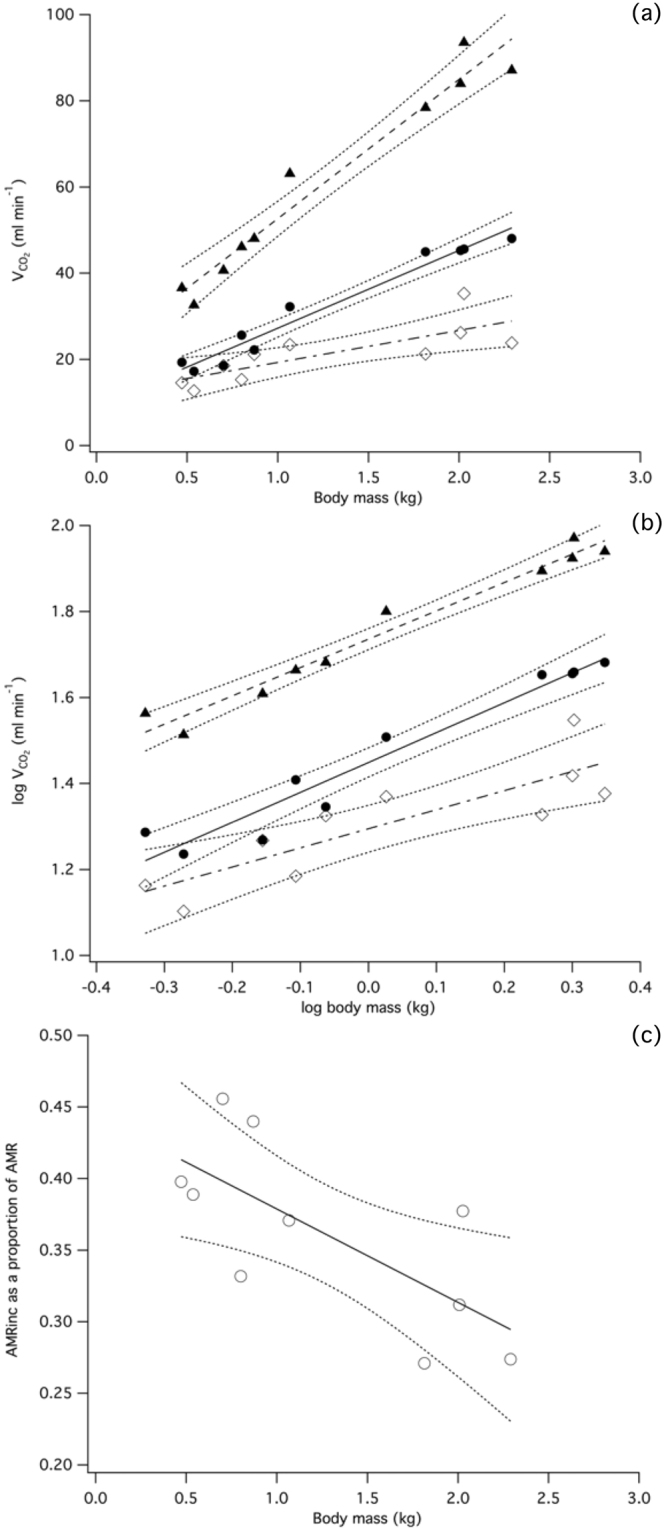
AMR (filled triangles), RMR_sit_ filled circles) and AMR_inc_ (open diamonds) over development, calculated per bird. (**a**) Development of RMRsit, AMRinc and AMR. Regression line equations: Sit: y = 18.072x + 9.152; P < 0.001; AMR_inc_: y = 7.428x + 11.864; P = 0.008; AMR: y = 32.330x + 20.319; P < 0.001). (**b**) Log-transformed data showing negatively allometric growth of AMR (y = 1.736x^0.660^, P < 0.001), i.e. per unit mass, metabolic rate declines as body mass increases. AMR_inc_ (y = 1.295x^0.444^, P = 0.002) and RMR_sit_ have negatively allometric growth (y = 1.449x^0.696^, P < 0.001). (**c**) Proportion of AMR accounted for by AMRinc over development. There is an inverse relationship between AMRinc and increasing body mass (y = −0.065x + 0.444; P = 0.021).

There was a positive relationship between increasing magnitude of the constituent elements of AMR and body mass (Fig. 3a, Table 1). Regression of log-transformed data (Fig. 3b, Table 1) highlighted allometric growth of AMR (∝Mb^0.66^) over development, i.e. per unit mass, metabolic rate declined as body mass increased. AMR_inc_ (∝Mb^0.44^) and RMR_sit_ (∝Mb^0.70^) also exhibited allometric growth, whereas the slopes of these regression lines diverged (ANCOVA: F = 4.791, P = 0.044). Plotting AMR_inc_ as a proportion of overall AMR (Fig. 3c), Table 1 highlighted the significantly reducing contribution of energy expended during exercise to AMR. The scaling trajectory of RMR_sit_ measured during group-respirometry did not differ significantly from the relationship derived independently in our study of postural RMR (ANCOVA: F = 0.3406, P = 0.561), although the intercept was increased in the group-based data (F = 26.91, P < 0.001).

**Figure 4:**
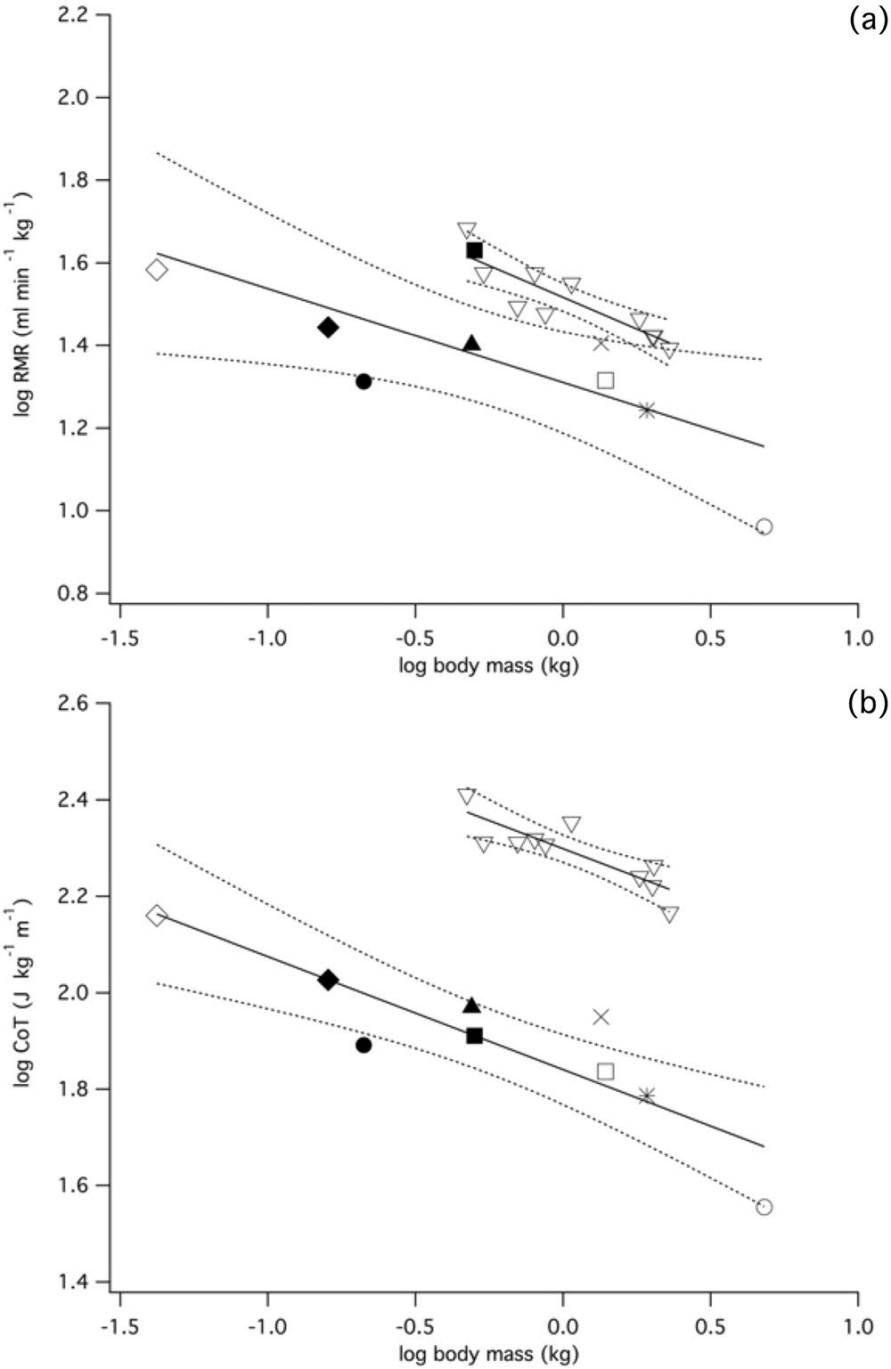
Comparative scaling of RMR and AMR variables in broilers and galliforms. (**a**) Scaling of RMR (V˙O_2_: ml min^−1^) in broilers (RMR_sit_: y = 1.517x^−0.307^; P = 0.001) and wild-type galliforms (intercept value at 0 ms^−1^: y = 1.310x^−0.227^; P = 0.026). (**b**) Scaling of CoT for broilers (open triangles: y = 2.299x^−0.234^; P = 0.002) walking at preferred speed and other galliforms (projected CoT at mean broiler walking speed 0.106 ms-^1^: y = 1.841x^−0.234^; P = 0.002). Symbols: painted quail (open diamond), bobwhite quail (filled diamond), chukar partridge (filled triangle), guinea fowl (cross) wild turkey (open circle):^68^; mountain quail (filled circle):^74^; Svalbard ptarmigan (filled square):^75^; leghorn bantam (open square), leghorn (asterisk):^76^.

Comparison of the scaling relationship between RMR and body size in broilers (RMR_sit_) and other galliforms (estimated RMR at 0 ms^−1^) indicates that the regression slopes do not differ (Fig. 4a, Table 2; ANCOVA: F = 0.2939, P = 0.5957) but the broiler intercept is significantly higher (ANCOVA: F = 17.85, P < 0.001). Hence across development, broiler RMR is elevated compared to equivalent-sized wild-type galliform species. Across the size range studied, broiler CoT follows a negative relationship with increasing body mass (Fig. 4b, Table 2), indicating that locomotion becomes energetically cheaper on a mass-specific basis in larger birds. Surprisingly, broiler and other galliform CoT regression lines are parallel (∝Mb^−0.23^: ANCOVA: F < 0.001, P = 0.993) although in contrast, the y-intercept for broilers is significantly higher (F = 230.2, P < 0.001). This indicates that broiler locomotion is inherently expensive at their preferred walking speeds compared to wild-type galliforms, regardless of developmental stage.

**Table 2:** Results of the regression analyses performed on broiler and galliform RMR (V˙O_2_ ml min^−1^ kg^−1^) and CoT (J kg^−1^ m^−1^).

	y-variable	N	F	t	R2	P	intercept	slope	slope 95% CI
log RMR	broiler	10	26.14	−5.113	0.766	0.001	1.517	−0.307	−0.446–0.169
	galliform	9	7.971	−2.823	0.532	0.026	1.31	−0.227	−0.417–0.037
log CoT	broiler	10	21.566	−4.644	0.729	0.002	2.299	−0.234	−0.350–0.118
	galliform	9	24.187	−4.918	0.776	0.002	1.841	−0.234	−0.347–0.122

## Discussion

The modern broiler chicken undergoes a remarkable morphological and physiological transformation in the 6-week hatch-to-slaughter period. We have demonstrated that behavioural and energetic aspects of broiler biology are associated with their rapid growth. Using our novel approach for determining metabolic rate, we fulfil our objective to provide the first detailed report of locomotor energetics across development in these commercially important birds. Intriguingly, broilers have a highly regulated total energy budget, to which resting metabolism accounts for a progressively greater proportion over development that limits energy available for other activities and likely contributes to a decline in locomotor capacity. Therefore we provided evidence to support our hypothesis of decreasing activity being associated with increasing metabolic costs during rest and locomotion.

The pronounced decline in the time spent actively moving over broiler development is in accordance with previous reports of behavioural changes as they age^25,39–41^. Activity declined from 12% in 2-week old chicks to below 5% in slaughter-weight birds. Birds observed in a commercial setting were found to follow a slightly different trajectory, in which activity declined at a slower rate initially (Fig. 1a). This difference may be explained by disparities between laboratory and farm-specific husbandry practices. For example, activity in broilers is influenced by light intensity^42^ and substrate material^43^, factors that were not exactly replicated in the laboratory. An experimental approach whereby all aspects of chick rearing are standardised could provide a better comparison. Walking speeds declined as the birds grew and followed a pattern similar to that observed for activity; i.e. steep decline until body mass reached a threshold value, followed by no further change. Mean and peak walking speed declined by around 20% and 260%, respectively, as body mass increased up to this breakpoint.

Our results are considerably lower than previous reports of broiler walking speed; for example voluntary walking at 1.01 ms^−1^ has been reported for 1.84 kg broilers^44^, although this is suggested to be the result of experimental design, i.e. broilers were tempted to walk towards a food reward after a period of feed withdrawal. Our results more closely parallel the slower speeds of 0.153–0.409 ms^−1^ ^45^ and 0.34 ms^−1^ ^5^ recorded for 6-week old broilers. Paxton^5^ reported top speeds in the region of between 0.6 and 1.1 ms^−1^, but again these are faster than those calculated from our video analyses. The fact that our estimates of walking speed were self-selected from free-ranging birds might explain the relatively slow speeds. Our experimental set up with the birds free to move to water or feeding dishes also more closely represented the conditions under which these birds are housed in the farms. Corr^45^ and Paxton^5^ derived walking speed from individuals encouraged to walk over a force plate. Encouraging the birds to move rather than allowing them to voluntarily chose the speed they move at could potentially result in faster speeds. Furthermore, our data were based upon an average of every bout of walking throughout the experimental period, regardless of the motivation to move. This averaging of the speeds to determine a mean value is useful for representing and matching to the simultaneous measurements of their locomotor energetics. Paxton^5^ mentioned that 65% of their data were excluded from analysis due to the slow and halting nature of walking broilers. While it is necessary to attain steady-state locomotion for biomechanical gait analysis^5,45^, discarding so many data points highlights the advantage of using an average speed over a given period of time (as we used here) to better quantify behavioural locomotor patterns and how these relate to energetics. We consider our approach to provide a more meaningful representation of the walking speeds used by broilers over development, in a ‘natural’ setting.

As broilers grow, standing becomes progressively more energetically costly. There is negatively allometric growth in RMR for sitting (∝Mb^0.77^) and standing (∝Mb^0.87^), which closely compares with reports of basal metabolic rate (BMR) (∝Mb^0.70^:^46^ and RMR, ∝Mb^0.80^:^47^ in developing broilers; i.e. there is an inverse relationship between mass-specific RMR and body mass. The result for sitting RMR also parallels the numerous analyses of interspecific basal metabolic rate in adult birds (∝Mb^0.67^ ^48,49^; ∝Mb^0.72^ ^50^ ∝Mb^0.74^ ^51^). Although details of the experimental protocols for measuring BMR more often than not omit information on posture, it is assumed that the birds will be sitting since this is posture is normally associated with quiet resting. Postural-specific RMR in mature birds has been reported in laying hens^52^ guillemots^53,54^, barnacle geese^55^ and guinea fowl^56^, and in each case, standing is associated with elevated RMR.

The energetic contribution of breathing mechanics to overall RMR may be influenced by the disproportionate growth of the breast muscles in broilers since active muscle contractions are required to power respiratory movements of the skeleton throughout the breathing cycle^23^. Dorsoventral movements of the sternum are required to maintain breathing in the standing bird, potentially contributing to an increased cost, notwithstanding the increase in postural muscle activity required to maintain balance in the larger bird as a result of a changing centre of mass (CoM)^4,5^. Broiler pectoralis major and minor (i.e. pectoralis and supracoracoideus) follow a positively allometric growth trajectory throughout the developmental period^11,14,15^, corresponding to the divergence in sitting and standing RMR. Due to previous studies^52–56^ considering only adult birds, we are unable to place our findings of the effect of posture on energetics in the context of what may be considered ‘typical’ for similar wild-type species. We hypothesise that the cost of breathing accounts for a fraction of RMR in proportion to breast muscle hypertrophy, although this remains to be fully tested. Indirect evidence for this hypothesis comes from research into artificial loading of the sternum, which greatly increases metabolic costs in birds^16,17^. Since most birds undergo pectoralis hypertrophy during development, it is likely that a similar relationship is present in other species although the magnitude of the energetic cost is likely to differ according to morphology and sternal mass.

The proportional difference of 39% between sitting and standing RMR in 3 kg broilers is the highest recorded, comparing with a 25% RMR disparity in barnacle geese^55^ and 16% for laying hens^52^. Interestingly, the proportion of body mass allocated to pectoralis major and minor is very similar in slaughter-weight broilers (17.6%^15^ and barnacle geese (17.8%:^57^, indicating that the relationship between breathing costs and sternal mass is determined by numerous biomechanical and physiological factors. For example, skeletal respiratory structures, the uncinate processes, are correlated with specialisation to primary locomotor mode in birds^20,58^. Terrestrial birds, including chickens, have relatively short uncinate processes and sternum^20^, meaning that there is potential for imposition of a functional limitation on breathing as development of large breast muscle mass on a skeleton that is optimised (in terms of breathing) for lighter muscle loads. Using the equation derived for broiler RMR, we estimate that RMR_stand_ for laying hens (Mb = 1.55 kg; c.f. van Kampen 1976) will be 25% higher than RMR_sit_; in fact the actual measured value is only 16%^52^. This disparity may in part be explained by the relatively small breast muscle mass in laying hens, accounting for only 12.3% of body mass, rather than 16.7% calculated for an equivalent broiler^15^. Coupled with the relatively slow growth compared to pectoral development of a primary expiratory muscle (i.e. muscle that elevates the sternal mass dorsally), the external oblique^15^, there is mounting evidence for the costly impact of selection for increasing breast muscle yields on breathing mechanics and energetics in broiler chickens.

Here we have highlighted the interaction of RMR and AMR in broiler chickens across their development. Broilers have a constrained energetic budget of which RMR progressively accounts for an increasing proportion (Fig. 3a,b, Table 1). When considered together with the results from the behavioural monitoring, it appears that decline in exercise duration and performance coincide with a reduction in the ‘expendable’ energetic resource. From the linear relationship between body mass and AMR (Fig. 3a, Table 1) we can infer that there is a functional energetic ceiling which the broiler is unwilling or unable to exceed. Coupled with the changing contribution of RMR and AMR_inc_, broiler energetics fit broadly into the allocation model of energetic resource partitioning^59^. Therefore, the scope for metabolic increase during activity is limited, and progressively reduces over the developmental period. This is a surprising finding given that access to food was not restricted, and is indicative of a highly regulated energy management. The relatively low level of activity in larger birds indicates that broiler exercise is restricted, and we hypothesise that this is in part due to an energetic constraint whereby the available metabolic resources are restricted within a defined overall energy budget. This trade-off between resting and activity metabolism has been experimentally determined in other avian species^60,61^, although a different energy management strategy, whereby resting and activity metabolic rates are independent of each other, has been observed in breeding gannets^62^. Our objective to characterise broiler energetics under ‘natural’ free-ranging conditions leaves unresolved the relationship between basal and maximal metabolic rate, which we did not quantify. Therefore it remains to be determined if the broiler has the metabolic capacity, or scope, to power extended bouts of activity or whether exercise performance is ultimately limited by, for example, deteriorating cardio-respiratory function or musculoskeletal pathologies.

It is likely that a complex interaction of factors including total activity, speed and postural-RMR determine the progressive decline in available energy for activity in the broiler. Our finding that the scaling slope for AMR is significantly shallower than for RMR parallels the result of a large-scale interspecific analysis^49^. Furthermore, the scaling exponent for broiler AMR (∝Mb^0.66^) closely matches that previously reported for meta-analyses of AMR (∝Mb^0.65^ ^49^; ∝Mb^0.71^ ^63^). Although developmental factors (e.g. growth rate, precocity etc.) may affect metabolic rate, it is striking that this relationship with body mass may be observed within a species over ontogeny, as well as interspecifically. Interestingly, a similar pattern of divergence between the developmental trajectories of RMR and AMR is present in jungle fowl (RMR ∝Mb^0.74^; AMR ∝Mb^0.52^ ^64^), indicating that the factors determining energy use may be conserved from the ancestral wild-type bird in the modern broiler. Taking into account a broader range of species highlights considerable variation in the scaling of juvenile RMR. For example RMR in terns^65^ and and Adelie penguin^66^ are reported to scale in excess of ∝Mb^1.00^, while albatross RMR develops ∝Mb^0.54^ ^67^. Therefore while the post-hatch scaling of broiler RMR is within the range measured for other species, RMR during the growing period in birds is affected by phylogenetic as well as environmental and morphological factors.

Fedak^68^ observed that at slower walking speeds, the proportion of active metabolic rate accounted for by RMR in birds will increase. Applying this principle to growing broilers, we expect to find increased proportional RMR as walking speed declines with body mass increase; while mean walking speed declines by 15% over development (Fig. 1b), AMR_inc_: RMR falls by approximately 25%. Following our rationale for calculating AMR_inc_, we can deduce a general *interspecific* AMR_inc_ using established relationships for RMR and AMR^49^. A far greater proportion of total energetic resources are available for activity in wild birds compared to broilers; for example, in a 2.5 kg wild bird and broiler, AMR_inc_ is equivalent to 1.47x RMR and 0.38x RMR, respectively. Although this difference is considerable, interspecific data were derived from studies of basal metabolic rate (i.e. unfed, inactive and in the dark), which differs from our determination of RMR. This methodological difference will cause the BMR intercept to be lower when compared to RMR because, by definition, additional costs such as digestion and thermoregulation must be borne by birds not in the basal state^49^. Broilers were also undergoing rapid bone, muscle and fat growth, likely causing elevated underlying resting costs^64^. No consideration of resting posture was included in the interspecific analysis, which, given our findings, may confound results on a mass-specific basis. A clear objective for future research should be to obtain ecologically relevant data that integrate behavioural and energetic aspects to resolve the intraspecific relationship between RMR and AMR.

Interspecific comparison of RMR indicates that even during periods of inactivity, broiler metabolic rate is elevated compared to other similarly sized galliform species (Fig. 4a, Table 2). This coincides with previous reports of increased metabolic activity being associated with selection for rapid growth and considerable deposition of protein in growing muscles^27^. We have also characterised the changing locomotor economy of broilers over development and demonstrated that larger birds have a reduced mass-specific energetic cost to move a unit distance (Fig. 4b). This negatively allometric trajectory (∝Mb^−0.23^) closely matches previous predictions (∝Mb^−0.25^:^69^; (∝Mb^−0.31^ ^70^ (galliforms ∝Mb^−0.23^) based on an interspecific size range of adult birds. Considering the progressive decline in broiler locomotor performance that is associated with increasing body mass, it is surprising that, at their preferred walking speeds, the slope of the line for CoT does not differ from projected CoT in comparably sized galliform species (Fig. 4b). In contrast, the y-intercept is significantly higher in broilers, indicating that walking is inherently costlier across all ages. The magnitude of the difference between AMR regression lines is not explained entirely by elevated broiler RMR (Fig. 4a), from which we can infer that locomotor activity (i.e. AMR_inc_) is relatively expensive. A similar conclusion was drawn in a comparison with jungle fowl^36^, whereby activity costs were assumed to be relatively high perhaps as a consequence of increased digestion costs in the broiler.

Despite high AMR, the mechanical cost of transport in broilers is approximately equivalent to other avian species^5^, suggesting that other factors contribute to an uneconomical gait. An absence of prominent energy-exchanging pendulum gait mechanics is predicted to cause increased locomotor cost^5^, while maintenance of stability by lateral movements of the legs during locomotion^71^ may contribute to elevated AMR_inc_. Development of broiler thigh and foot segments with relatively high mass may also impart an unfavourable metabolic penalty during locomotion due to relatively large moments of inertia^4^. In all other comparably sized galliforms, slow walking is associated with relatively higher energetic expenditure for each step taken, thereby imposing an energetic ‘penalty’ on growing broilers that are constrained to uneconomical speeds (Fig. 1b). Therefore, the principal differences between other galliforms and broilers are not found in the scaling of the energetics of locomotion *per se;* instead, broilers are challenged by a combination of uneconomical CoT and constraint on walking speed.

Why voluntary walking speed in the broiler should be suboptimal in energetic terms can be explained by numerous factors including maintenance of stability^2^, development of pathological conditions^4^ and changing body morphology. Broilers are susceptible to pathological conditions of the hindlimbs that eventually cause lameness, potentially due in part to the development of relatively high body mass on an immature skeleton^2,21^. Although care was taken to ensure each bird had a normal gait, it is possible that underlying leg pathologies may have influenced preferred walking speed. For example, skeletal abnormalities such as tibial dyschondroplasia and bacterial chondronecrosis may occur across the developmental period, but these conditions do not necessarily affect normal gait^4,72^ Conversely, there may be an explanation for slow walking speed that is related to overall energy expenditure. Given that total metabolic rate (i.e. whole-animal V˙O_2_ rather than V˙O_2_ per unit distance) and walking speed are positively correlated in broilers, by limiting speed the absolute energetic resources required for locomotion are minimised. Movement across relatively small distances coupled with a large proportion of time spent inactive suggests that there is only a modest penalty when using inefficient slow speeds on the overall energy budget. As additional heat is produced with increasing metabolic effort, a potential advantage of limiting walking speed (and therefore metabolic rate) is to reduce heat stress on the birds. Effective insulation improves with feather development while the surface area to volume ratio decreases as the birds grow. Combined these factors means that excess metabolic heat becomes relatively more difficult to disperse, which corresponds with decreased activity (Fig. 1a). Close regulation of ambient temperature and ventilation is required for adequate heat dissipation in broilers^73^, indicating that substantial increases in metabolic rate during locomotion are undesirable.

When we consider the welfare implications of this system, the declining energetic resources made available for movement coupled with a restricted speed range highlight how exercise performance is compromised in the broiler. We have demonstrated that activity levels and metabolic costs are inversely related over development, resulting in the heaviest birds exhibiting relatively low activity levels, potentially resulting from their energetically expensive locomotion. Our integrative approach for assessing the interactions between behaviour and physiology has highlighted potential targets for selection that may help to produce healthier birds, for example improving the musculoskeletal respiratory apparatus. Our results represent a stepping-stone towards deciphering the complex trade-offs between growth rate, pectoral mass and metabolic physiology, and how these factors contribute to behavioural changes. Future breeding programs can build on this evidence to produce healthier birds, bringing benefits to welfare and economic aspects of production.

### Ethics

The study was approved by the Animal Use Ethics Committee at the University of Manchester and conducted under Home Office licence 40/3549.

### Data Accessibility

The datasets used for this manuscript have been uploaded as part of the supplementary material.

## Electronic supplementary material

Dataset 1
